# Genome-forward oncology: how do we get there?

**DOI:** 10.1186/gb-2011-12-s1-i5

**Published:** 2011-09-19

**Authors:** Matthew Ellis

**Affiliations:** 1 Breast Cancer Program and the Genome Institute, Washington University in St. Louis, St. Louis, MO, USA

## 

Massively parallel sequencing is transforming our knowledge of cancer, yet the medical value of next-generation approaches has not been fully established. From a technical perspective, it is easy to envisage that, within a few years, the primary diagnostic approach for all cancers will be to assess a partial or whole cancer genome sequence; however, the adoption of this approach will ultimately depend on the development of robust and valid models for the tailoring of therapy. Thus, within a short period, the focus of genomic investigation will shift from the current emphasis on discovery in poorly annotated datasets, such as The Cancer Genome Atlas, to ambitious investigations that focus on precise clinical questions. This transition will occur in two stages. The first stage will be a retrospective, ‘genome-backward’ approach, in which patients are treated blind to genomics but consent to prospective germline and tumor sequencing, as well as data sharing. In this way, models that use mutation patterns to predict treatment outcomes can be developed. In a later prospective, ‘genome-forward’ phase, therapeutic postulates that arise from genome sequencing will be used as the basis for clinical trial eligibility or stratification. Specific examples of how these approaches are being studied in breast cancer will be discussed.

